# Availability and Primary Health Care Orientation of Dementia-Related Services in Rural Saskatchewan, Canada

**DOI:** 10.1080/01621424.2015.1092907

**Published:** 2015-12-14

**Authors:** Debra G. Morgan, Julie G. Kosteniuk, Norma J. Stewart, Megan E. O’Connell, Andrew Kirk, Margaret Crossley, Vanina Dal Bello-Haas, Dorothy Forbes, Anthea Innes

**Affiliations:** ^a^ Canadian Centre for Health and Safety in Agriculture, University of Saskatchewan, Saskatoon, Saskatchewan, Canada; ^b^ College of Nursing, University of Saskatchewan, Saskatoon, Saskatchewan, Canada; ^c^ Department of Psychology, University of Saskatchewan, Saskatoon, Saskatchewan, Canada; ^d^ Division of Neurology, College of Medicine, University of Saskatchewan, Saskatoon, Saskatchewan, Canada; ^e^ Department of Psychology (Professor Emerita), University of Saskatchewan, Saskatoon, Saskatchewan, Canada; ^f^ School of Rehabilitation Science, McMaster University, Hamilton, Ontario, Canada; ^g^ Faculty of Nursing, University of Alberta, Edmonton, Alberta, Canada; ^h^ University of the West of Scotland, Hamilton, Scotland

**Keywords:** access, availability, dementia, primary health care, rural health service delivery

## Abstract

Community-based services are important for improving outcomes for individuals with dementia and their caregivers. This study examined: (a) availability of rural dementia-related services in the Canadian province of Saskatchewan, and (b) orientation of services toward six key attributes of primary health care (i.e., information/education, accessibility, population orientation, coordinated care, comprehensiveness, quality of care). Data were collected from 71 rural Home Care Assessors via cross-sectional survey. Basic health services were available in most communities (e.g., pharmacists, family physicians, palliative care, adult day programs, home care, long-term care facilities). Dementia-specific services typically were unavailable (e.g., health promotion, counseling, caregiver support groups, transportation, week-end/night respite). Mean scores on the primary health care orientation scales were low (range 12.4 to 17.5/25). Specific services to address needs of rural individuals with dementia and their caregivers are limited in availability and fit with primary health care attributes.

## INTRODUCTION

In 2012 the World Health Organization (WHO) declared dementia a public health priority and called on governments, policy makers, and other stakeholders to respond to this growing challenge to global health (WHO and Alzheimer’s Disease International [ADI], 2012). The increasing impact of dementia has led many countries to identify dementia as a national priority. A common focus across national dementia strategies is access to information, support, and services for individuals with dementia and their families across the care continuum (ADI, 2012; WHO, 2012). Compared with other chronic conditions, individuals with dementia need more personal care and supervision, with associated greater caregiver strain and costs (ADI, 2013). A comprehensive system of care for people with dementia encompasses both health and social services (Moise, Schwarzinger, & Um, 2004), from diagnostic to end-of-life care (ADI, 2013). Core principles underlying planning for dementia include delaying institutionalization by supporting caregivers in maintaining home-based care, local coordination of services, and matching services to need (Moise et al., 2004).

Community-based services contribute to improved outcomes and quality of life for individuals with dementia and their caregivers, yet providing access can be challenging in rural and remote areas. Geographic isolation, small populations, and limited access to health professionals are barriers to delivery of appropriate health services (Bourke, Humphreys, Wakerman, & Taylor, 2012), despite the fact that rural areas are often sites of innovation in health service delivery (Bourke, Humphreys, Wakerman, & Taylor, 2010). Canadian seniors account for a greater share of the rural than urban population (Dandy & Bollman, 2008), thus rural communities have a higher proportion of individuals at risk for dementia, but often less capacity to provide services. Out-migration of younger, working-age cohorts has contributed to the aging of rural communities and reduced the informal support networks needed to maintain seniors’ independence (Skinner, Hanlon, & Halseth, 2012).

Previous research has identified the challenges of rural health service delivery for people with dementia. Parallel systematic reviews examining informal (Innes, Morgan, & Kosteniuk, 2011) and formal (Morgan, Innes, & Kosteniuk, 2011) caregiving for dementia in rural settings identified the need for dementia-specific education for health care providers, and development of coordinated models of service delivery. A review of diagnosis and postdiagnostic support for dementia in rural areas (Szymczynska, Innes, Mason, & Stark, 2011) found that services were often difficult to access due to transportation difficulties and distance from specialist services. Caregivers of individuals diagnosed with dementia at a rural and remote memory clinic (Morgan, Walls-Ingram, et al., 2014) frequently reported challenges in obtaining a diagnosis from their primary care provider, which was perceived as a barrier to accessing services.

Strengthening local primary health care systems has been identified as a key strategy for managing changing demographics population aging and the increasing prevalence of chronic diseases (Wakerman, 2009). Primary health care includes primary prevention, public health, and health care services that are provided by a range of providers in a variety of settings, in a way that is person- and population-centered (Canadian Institutes of Health Research [CIHR], 2014). Comprehensive primary health care services are key to improving dementia care (Aminzadeh, Molnar, Dalziel, & Ayotte, 2012), particularly in rural and remote communities because of the focus on responsiveness to the local context and equity in health outcomes (Buykx et al., 2012; Wakerman, 2009). More research is needed to build innovative and sustainable primary health care models that are appropriate to the rural context (Wakerman, 2009).

Although it is evident that there are challenges in rural health service delivery for dementia, there are few comprehensive assessments of availability and quality of dementia services. The purpose of this needs assessment study was to identify strengths and gaps in rural dementia care by determining (a) the availability of rural dementia-related services across the continuum of care within all of the 13 health regions of the Canadian province of Saskatchewan, and (b) the orientation of rural dementia-related services toward key aspects of primary health care. This research contributes to the knowledge base about dementia-related service availability and quality in rural settings, and provides evidence for policy and program development to address identified needs and gaps.

## METHOD

### Background and Design

Data were collected using a cross-sectional survey of Home Care Assessor/Coordinators in the western Canadian province of Saskatchewan. The study is part of a larger project involving a comparison of actual to best practices in dementia care (gap analysis) to identify priorities for policy, knowledge exchange, and research, and to provide baseline data for a longitudinal study aimed at developing new models of rural primary health care for dementia (Morgan, Kosteniuk, et al., 2011; Morgan, Crossley, et al., 2014; Kosteniuk et al., 2015). The research was guided by a Steering Committee comprised of the Alzheimer Society of Saskatchewan, health region leadership, and health care professionals.

### Setting

**FIGURE 1:**
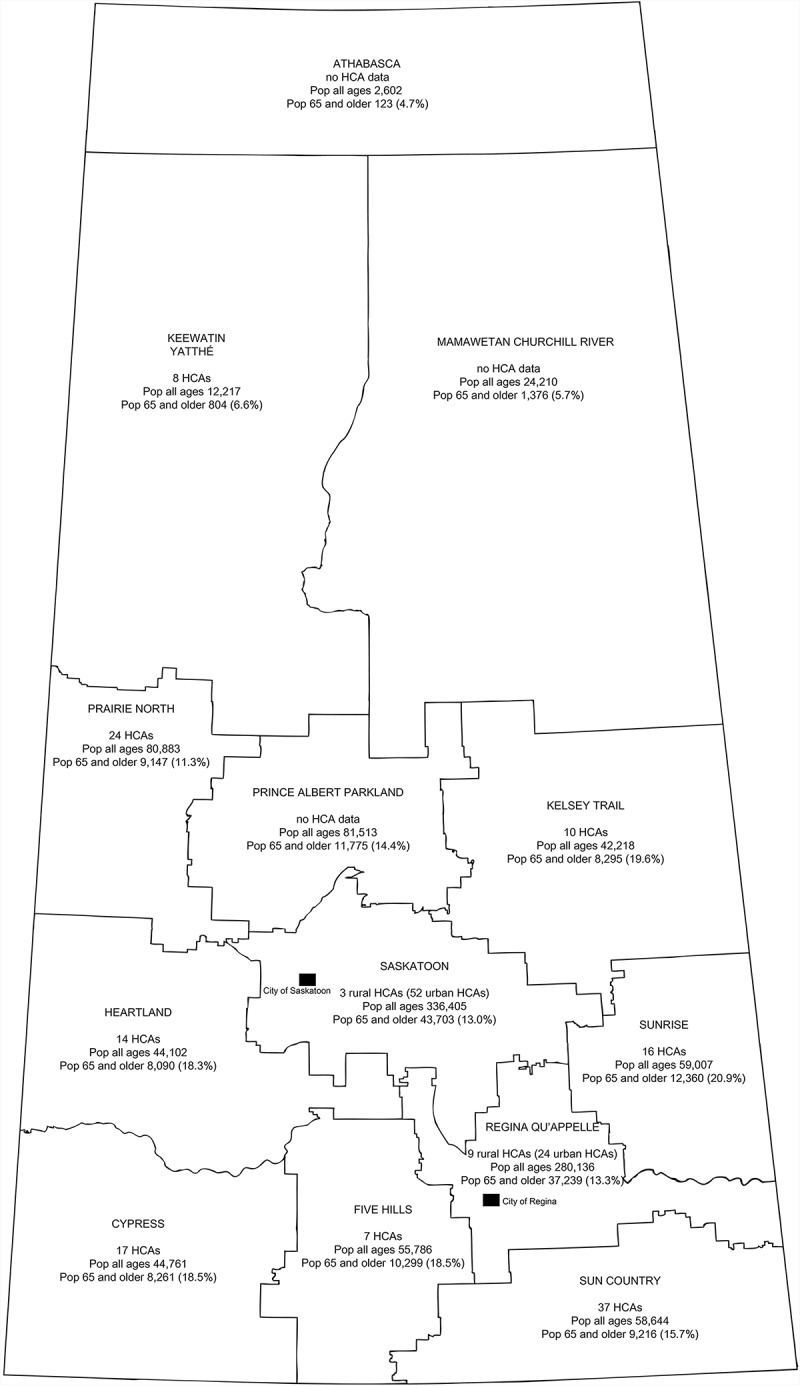
Number of home care assessors, based on query to home care directors, and population counts, by Health Region, Saskatchewan, 2013 (Saskatchewan Ministry of Health, 2013).

The province of Saskatchewan (Figure 1) spans over 650,000 km^2^ and has a population of 1.1 million people. The mean population density is 1.8 versus 3.7 persons/km^2^ for Canada (Statistics Canada, 2012a). Sixty percent (60%) of residents live outside the province’s two largest cities (census metropolitan areas), specifically Saskatoon (population = 222,289) and Regina (population = 193,100; Saskatchewan Bureau of Statistics, 2011). Areas outside of these major centers are defined as rural for this article. Approximately 160,000 individuals aged ≥ 65 account for 14.3% of the Saskatchewan population (Statistics Canada, 2012b). Across the 13 health regions, the proportion of seniors varies from 4.7 to 20.9%, with a higher proportion in smaller centers such as towns (21.3%), villages (20.1%), and recreational villages (25.8) compared to cities (14%; Elliot, 2012). The Saskatchewan health system has a regional model of governance with each health region responsible for providing basic health services (Saskatchewan Ministry of Health, n.d.).

### Sample

The study population consisted of all Home Care Assessors serving the 60% of Saskatchewan residents living outside of the two major cities (rural Assessors). The Home Care Program in Saskatchewan is administered and delivered by regional health authorities and funded by the provincial Ministry of Health. Home Care Assessors were identified by the study Steering Committee as ideal informants because they are knowledgeable about needs of individuals with dementia and the services in their local communities. Assessors (health region employees who are registered nurses or social workers) provide single point of entry to regional home care services, which are aimed at maintaining independence and well-being at home. Their role involves assessment and coordination of home care services, case management, and making referrals to other appropriate community agencies and long-term care. To establish the number of Assessors in each region, in May 2013, an initial email query was sent to the Director of Home Care for each of the 13 health regions. The email included a notice about the survey and a request for feedback regarding the number of Assessors serving each geographic area. It was determined that there were approximately 145 rural Assessors. Eligible participants were defined as those currently employed as rural Home Care Assessors in a Saskatchewan health region. Ethics approval was granted by the University of Saskatchewan and Sun County Health Region Health Region Research Ethics Board (BEH 12–341) as well as the ethics committees of health regions requiring regional-level approval.

### Data Collection

#### Survey Instrument and Development Process

The study Steering Committee provided expert review of the first iteration of the questionnaire in May 2013, regarding the clarity of instructions and measures, and whether any items needed to be added or removed. The second iteration was piloted with three Assessors in one health region. We incorporated their recommendations and asked them to review a third iteration. The fourth iteration of the survey was used in this study.

The survey consisted of two parts. Part A presented respondents with 43 dementia-related services and resources that covered five areas along the continuum of care (i.e., health promotion programs, primary health care, postdiagnosis support, home care, and long-term care). Part B consisted of 30 items covering six key dimensions of primary health care associated with dementia-related services.

##### Availability of Dementia-Related Services Across the Continuum of Care (Part A)

The lead researcher (DM) hosts an annual knowledge exchange meeting of stakeholders known as the Summit of the Knowledge Network in Rural and Remote Dementia Care (Morgan, Crossley, et al., 2014). At the 2012 event, we conducted five focus groups to brainstorm a list of ideal dementia-related services. The groups were provided with a checklist of several ideal services from prediagnosis to end-of-life care, identified from national dementia strategies (Cahill, O’Shea, & Pierce, 2012; Department of Health, 2009; Dudgeon, 2010; Health Foundation, 2011) and asked to identify additional ideal services. These data were compiled to create a final list of 43 services that formed Part A. Of these services, 13 were dementia specific (e.g., dementia support group), while the remainder were more basic services (e.g., home care services such as personal care and homemaking services) that could be helpful to individuals with dementia and their caregivers but were not designed specifically for dementia.

Part A asked respondents to “indicate the availability of services in the *majority of communities* for which you are primarily responsible.” Since Assessors served multiple communities, this instruction (recommended by participants in the pilot phase) meant that Assessors would not be required to choose one community on which to base their answer. Responses included “everyday,” “a few times a week,” “once a week,” “once a month,” “less often than once a month,” and “not available.” For the present analysis the responses were grouped into “at least weekly,” “less often than weekly,” and “not available.” For the housing options (personal care home, nursing home, assisted living, seniors housing, special care units), availability was grouped into “available” or “not available.”

##### Primary Health Care Orientation of Dementia-Related Services (Part B)

The quality of existing dementia services across the care continuum in Saskatchewan health regions was assessed with six 5–item scales developed to measure the degree to which Assessors believed current dementia-related services were consistent with the ideal attributes of primary health care delivery. Scale development was informed by two streams in the literature: (a) ideal dementia-related services and resources along the care continuum (Cahill et al., 2012; Department of Health, 2009; Dudgeon, 2010; Health Foundation, 2011), and (b) important dimensions of primary health care, as described by Levesque and colleagues (2011). Scale development was also informed by the “community-based primary health care” concept that primary health care is delivered in a range of settings and includes activities beyond medical care (CIHR, 2014). The six scales developed for the present study addressed six key aspects of primary health care: information and education, accessibility, population orientation (community fit), coordinated care, comprehensiveness of care, and quality of care. All Part B items were focused on dementia.

Respondents were asked to think of “community” as the “majority of communities for which you are primarily responsible in your health region.” Each of the six scales consisted of five items rated on a 5-point scale from 1 (*no, not at all*) to 5 (*yes, to a very great extent*), with total scale scores ranging from 5 to 25. The six scales were Information and Education, Accessibility, Population Orientation (fit with community needs), Coordinated Care, Comprehensiveness of Care, and Quality of Care. For each scale item (e.g., appropriate frequency of services), participants were asked to assess the degree to which the sum of available services in their community was aligned with a particular aspect of primary health care (e.g., Accessibility scale). A single item asked whether “the amount of supportive resources and services available in the community is adequate to allow individuals with dementia to remain at home for as long as possible,” with response similar to those for the scale items. Where a participant’s scale was missing 25% or less of the items (i.e., one item) (El-Masri & Fox-Wasylyshyn, 2005), the case mean was imputed. If more than 25% of the items (i.e., 2 or more) in a scale were missing for a participant, their score was not included in the overall mean for the scale. Higher scale scores indicated greater orientation to key aspects of primary health care.

##### Open-Ended Comments

Space for comments was provided after Part A and Part B to allow participants to elaborate on their responses in these two sections of the survey.

#### Data Collection Procedure

In June 2013, an email message was sent to the Director of Home Care of each region, with a survey package that included an invitation letter, consent form, and survey. Each Director was requested to forward the message to all Home Care Assessors in their region. The packages were not sent directly to Assessors because the names and contact information of health region employees are not in the public domain. The invitation letter requested that Assessors return their completed surveys to the project research coordinator by fax or email. Follow-up thank you and reminder emails were sent to Directors at 1 week, 2 weeks, 1 month, 2 months, and 4 months after initial contact, for a total of six contacts with the Directors, and potentially five contacts with each Assessor. Data collection took place June to December 2013.

### Data Analysis

All data were analyzed using SPSS 20.0 (SPSS Inc., Chicago, IL, USA). Descriptive statistics were used to investigate the availability of dementia-related services, including frequencies and proportions. Mean scores, standard deviations, and ranges were reported for each of the items in the six Primary Health Care Orientation scales and for each scale. Internal consistency reliability of each scale was assessed with Cronbach’s alpha, with a coefficient of ≥ .70 used to determine acceptable internal consistency reliability (Nunnally & Bernstein, 1994). The open-ended comments regarding availability and quality of dementia services were combined and themes were identified.

## RESULTS

Survey respondents included 71 Home Care Assessors. Three of the 13 health region Directors did not provide information regarding the number of Assessors serving their regions. Therefore, the estimated rate of response was 49% (71 of the known 145 Home Care Assessors). One or more Assessors from 12 regions returned a completed survey and are included in the study. One health region is not represented. Identifying information such as age and gender was not required to address the study aims and was therefore not requested as it may have discouraged respondents from participating. Respondents were asked to specify the geographic area(s) that they served and some areas may have been served by only one individual.

### Availability of Services (Part A)

As shown in Table 1, *health promotion programs* aimed at healthy lifestyle and brain health were reported to be widely unavailable according to the majority of respondents. The exception was recreational activities for older adults, which most respondents indicated were available at least weekly in their communities (81.7%; *n* = 58/71). *Primary health care* services that were available at least weekly according to more than half of respondents, included pharmacists (88.4%), family physicians (84.5%), physical therapists (62.3%), and occupational therapists (54.9%). Nurse practitioners were unavailable (52.1%) in just over half of communities served, as was multidisciplinary team assessment (57.1%). *Postdiagnostic support* services that most respondents indicated were available weekly included palliative care (91.5%), adult day programs (76.1%), and case management of diagnosed individuals (71.0%). Approximately half of respondents reported availability of volunteer services/visitors (51.4%). The majority of respondents reported that other postdiagnostic support was not available, including private caregiving (66.7%) and caregiver support groups (62.3%), and more than half reported no access to counseling for diagnosed individuals (54.9). *Home care services* that were widely available weekly included morning care (98.6%), bath assist (98.6%), meals on wheels and meal preparation (94.4%), toileting (84.3%), planned respite (69.6%), and in-home respite (76.1%). Home care services that were unavailable in most communities, according to the majority of respondents, included night respite (73.2%), weekend respite (51.4%), and transportation to health care (55.7%). With respect to *long-term care services*, counseling for individuals with dementia (73.8%) and caregivers (67.2%) was widely unavailable. Long-term care housing (data not reported in Table 1) available in the community, according to most respondents, included seniors housing (92.5%), nursing homes (76.6%), and private care homes (59.1%). Long-term housing that was more likely to be unavailable included assisted living (62.7%) and dementia special care units (60.9%).

**TABLE 1:** Availability of Dementia-Related Services

Service	*N*	At least weekly *n* (%)	Less often than weekly *n* (%)	Not available *n* (%)
Health promotion programs				
Recreational activities for older adults	71	58 (81.7)	9 (12.7)	4 (5.6)
Healthy lifestyle promotion related to dementia	70	8 (11.4)	10 (14.3)	52 (74.3)
Healthy brain promotion	69	5 (7.2)	10 (14.5)	54 (78.3)
Primary health care				
Pharmacist	69	61 (88.4)	3 (4.3)	5 (7.2)
Family physician	71	60 (84.5)	5 (7.0)	6 (8.5)
Physical therapist	69	43 (62.3)	17 (24.6)	9 (13.0)
Occupational therapist	71	39 (54.9)	30 (42.3)	2 (2.8)
Other health care professionals	69	34 (49.3)	14 (20.3)	21 (30.4)
Social worker	70	33 (47.1)	19 (27.1)	18 (25.7)
Nurse practitioner	71	30 (42.3)	4 (5.6)	37 (52.1)
Screening of older adults for possible dementia	69	24 (34.8)	15 (21.7)	30 (43.5)
Multidisciplinary team assessment	70	18 (25.7)	12 (17.1)	40 (57.1)
Postdiagnosis support				
Palliative care	71	65 (91.5)	4 (5.6)	2 (2.8)
Adult day program	71	54 (76.1)	2 (2.8)	15 (21.1)
Case management of diagnosed individuals	69	49 (71.0)	9 (13.0)	11 (15.9)
Volunteer services/visitors	70	36 (51.4)	13 (18.6)	21 (30.0)
Private caregiving	66	21 (31.8)	1 (1.5)	44 (66.7)
Other caregiver support	70	18 (25.7)	25 (35.7)	27 (38.6)
Counseling diagnosed individuals	71	15 (21.1)	17 (23.9)	39 (54.9)
Counseling caregivers	71	14 (19.7)	23 (32.4)	34 (47.9)
Patient registry (for individuals diagnosed with dementia)	69	6 (8.7)	5 (7.2)	58 (84.1)
Caregiver support group	69	3 (4.3)	23 (33.3)	43 (62.3)
Caregiver registry (for caregivers of individuals diagnosed with dementia)	65	1 (1.5)	3 (4.6)	61 (93.8)
Home care				
Personal care—AM care	71	70 (98.6)	1 (1.4)	0
Personal care—Bath assist	71	70 (98.6)	1 (1.4)	0
Meals on Wheels	71	67 (94.4)	0	4 (5.6)
Meal prep	71	67 (94.4)	1 (1.4)	3 (4.2)
Personal care—Toileting	70	59 (84.3)	1 (1.4)	10 (14.3)
Personal care—HS Care	71	56 (78.9)	1 (1.4)	14 (19.7)
In-home respite and visiting	71	54 (76.1)	10 (14.1)	7 (9.9)
Homemaking	70	52 (74.3)	3 (4.3)	15 (21.4)
Planned respite care	69	48 (69.6)	20 (29.0)	1 (1.4)
Emergency respite	71	31 (43.7)	12 (16.9)	28 (39.4)
Transportation to health care	70	27 (38.6)	4 (5.7)	39 (55.7)
Weekend respite	70	24 (34.3)	10 (14.3)	36 (51.4)
Night respite	71	14 (19.7)	5 (7.0)	52 (73.2)
Long-term care*				
Counseling for caregivers of individuals in LTC	64	10 (15.6)	11 (17.2)	43 (67.2)
Counseling for individuals with dementia in LTC	65	6 (9.2)	11 (16.9)	48 (73.8)

*Note. N* varies from 64 to 71 due to missing responses across items.

*Other long-term care services (seniors housing, nursing home, private care home, assisted living, dementia special care unit) are reported in the text because a different response set was used for these services.

### Primary Health Care Orientation of Dementia-Related Services (Part B)

Individual items were rated on a 5-point scale, with higher scale ratings indicating greater orientation of services to key aspects of primary health care. Mean scores on the *Information and Education* items (Table 2) ranged from strongly negative with respect to adequacy of public education to reduce stigma (1.8, *SD* = 0.8) to somewhat positive with respect to adequacy of dementia awareness among local health care professionals (3.5, *SD* = 1.2). Respondents also held somewhat negative perceptions of the *Accessibility* of dementia services, specifically regarding geographic location (2.2, *SD* = 1.2) and availability of subsidized/free transportation (2.0, *SD* = 1.3). Similarly, *Population Orientation* (i.e., community fit) was perceived as inappropriate on average, specifically with regard to level of caregiver support (2.1, *SD* = 0.9) and level of primary health care services (2.3, *SD* = 1.0). The dimension of *Coordinated Care* fared more favorably, with perceptions being neutral (2.9) on care transition as well as ease of access to patient health history, and somewhat positive on coordination of patient care and service delivery (3.2 to 3.6). Similarly, respondents held somewhat negative to somewhat positive views of *Quality of Care* with respect to timely diagnosis (2.7, *SD* = 0.9) and effectiveness of screening tools (3.4, *SD* = 0.8). For the single item “The amount of supportive resources and services available in the community is adequate to allow individuals with dementia to remain at home for as long as possible,” the mean was 2.6 (*SD* = 1.1, range 1–4, *n* = 69).

Table 3 reports Cronbach’s alpha scores for the six scales in Part B, ranging from .58 (*Accessibility*) to .78 (*Information and Education*). The mean score of the *Information and Education* scale of 12.7 (*SD* = 3.8, range = 6–23) demonstrates that respondents perceived community-level

**TABLE 2:** Primary Health Care Orientation of Dementia-Related Services Scales, Item Statistics

Attribute	*M*	*SD* (range)
Information and education		
Adequate awareness about “what to do” or “where to go”	2.7	1.2(1−5)
Adequate information for caregivers	2.3	1.0(1−4)
Adequate awareness of dementia among health care professionals in the community	3.5	1.2(1−5)
Adequate dementia-specific continuing education for health care professionals	2.4	1.0(1−5)
Adequate public education to reduce stigma of dementia	1.8	0.8(1−4)
Accessibility		
Appropriate frequency of services	2.5	1.1(1−5)
Appropriate wait time for services	2.8	1.2(1−5)
Available public transportation to services(for older adults)	2.9	1.5(1−5)
Available subsidized/free transportation to services(for older adults)	2.0	1.3(1−5)
Equally accessible services, regardless of geographic location	2.2	1.2(1−5)
Population orientation		
Appropriate level of PHC services	2.3	1.0(1−5)
Appropriate level of home care services	3.0	1.1(1−5)
Appropriate number of LTC beds	2.6	1.3(1−5)
Appropriate telehealth services	2.8	1.1(1−5)
Appropriate level of support for caregivers	2.1	0.9(1−4)
Coordinated care		
Service delivery by different health care professionals in the community is coordinated	3.4	1.1(1−5)
All health care professionals in the community have easy access to patient health history	2.9	1.3(1−5)
Health care professionals co-ordinate well with each other to manage patient care(within and outside community)	3.5	1.0(1−5)
Health care professionals co-ordinate well with community agencies to manage patient care(within and outside community)	3.2	1.0(1−5)
Seamless transition from community to LTC	2.9	1.2(1−5)
Comprehensiveness of care		
One or more health care professional is able to diagnose	3.3	1.4(1−5)
One or more health care professional is able to provide on-going management	3.9	0.8(2−5)
There is timely referral to appropriate health and social services	3.2	1.0(1−5)
Multidisciplinary care is available	3.6	1.1(1−5)
Health care professionals consider dementia a chronic disease	3.6	1.1(1−5)
Quality of care		
Timely diagnosis occurs	2.7	0.9(1−5)
Health care professionals use standardized diagnostic criteria	3.2	1.1(1−5)
Current screening tools are effective	3.4	0.8(1−5)
Care and management are guided by standardized care pathways	2.5	1.0(1−4)
Health care professionals adequately monitor safety of individuals with dementia living at home	2.9	1.1(1−5)

*Note. N* varies from 69 to 70 due to missing responses across items.

Scale scoring: 1 = no, not at all; 2 = no, not really; 3 = undecided; 4 = yes, to some extent; 5 = yes, to a very great extent.

**TABLE 3:** Primary Health Care Orientation of Dementia-Related Services Scales, Statistics, and Internal Consistency Reliability

Scale	*n*	*M* score (*SD*)	Range^a^	Cronbach’s alpha^b^
Information and education	70	12.7 (3.8)	6–23	0.78
Accessibility	69	12.4 (3.9)	6–22	0.58
Population orientation	70	12.7 (3.3)	5–21	0.60
Coordination of care	70	15.9 (3.6)	8–24	0.67
Comprehensiveness of care	70	17.5 (3.7)	7–25	0.74
Quality of care	70	14.5 (3.2)	8–21	0.69

*Note. N* varies from 69 to 70 due to missing responses across items.

^a^All scales have 5 items, rated on a 5-point scale (possible range 5–25).

^b^Cronbach’s alpha based on standardized items.

dementia-related information and education to be inadequate. Respondents reported overall negative perceptions of *Accessibility* and *Population Orientation* related to community dementia services, with respective average scale scores of 12.4 (*SD *= 3.9, range = 6–22) and 12.7 (*SD* = 3.3, range = 5–21). The overall *Coordinated Care* scale score was in the neutral range at 15.9 (*SD *= 3.6, range = 8–24). The *Comprehensiveness of Care* scale score of 17.5 (*SD* = 3.7, range = 7–25) reflects the somewhat positive position of respondents on this dimension. The *Quality of Care* scale score of 14.5 (*SD* = 3.2, range = 8–21) reflects the neutral position of respondents.

### Themes Identified in Open-Ended Comments

Four themes were identified in the comments made by participants at the end of Part A (availability of services) and Part B (primary health care orientation). Examples of comments are provided to illustrate these themes.

#### Limited Service Options in Rural Communities

Limited access and availability contributed to long waitlists and referrals to urban centers:

Most rural communities have limited Home Care services (e.g., one visit a day, Monday to Friday). Often limited access to physician, no support groups for caregivers. (FIV007)

Of the services that are available, the majority of the support has no room available and often people are waitlisted due to limited resources, i.e., Home Care/Respite/Adult Day Program/Special Care Home/OT/PT. (SAS004)

The questions above assume that there are services and multi-disciplinary professionals available in rural communities. They aren’t. We try to refer to resources in the city and we often don’t get a response or the response we do get is how busy they are and that they won’t be coming out for a very long time. (SAS003)

#### Limited Staffing and Funding for Services

Inadequate availability of services increased reliance on informal supports to allow people with dementia to continue to live at home:

Very limited resources and staffing make it very difficult to provide care that would be ideal for clients. A very large base of support is provided by Home Care (when available) or family support. (SAS004)

The services available are often dependent on staffing at the time especially for respite care in the home. Respite may be prebooked but is the first service to be cancelled if not enough staff. Also keeping dementia clients in the home is dependent on family and friend support. It’s difficult for the services to be adequate if this support isn’t there. (SUC009)

#### Negative Consequences Associated With Inadequate Levels of Services

Inability to meet the needs of people with dementia and their caregivers in the community were reported as risks for earlier long-term care placement and having to leave one’s home community to receive specialized services:

Home Care does what it can to keep people at home, but we could keep people out of institutional care longer if we had these things. People don’t want to leave this community to find these services elsewhere. (HEA009)

Most available professional services are part time so it is difficult to provide a good team approach—no long-term care bed/room specifically for dementia patients. All residents of the community over the past 5 years have had to move out of the community for long-term care placement. (SUC002)

#### Late Referrals for Services, When Dementia Is Advanced or During a Crisis

Participants stated that earlier diagnosis and referral, providing information about available services to persons with dementia and their families, and better availability of resources might help to avoid crises and delay institutionalization:

Families in rural communities deal with family members with dementia without much support available for long periods of time. Often the dementia is very advanced prior to families seeking help and that is due a lot to the limited resources in rural communities. (SUC004)

There are many primary caregivers of family members with dementia who do not seek help until a crisis occurs, often the support that could have been arranged sooner may have assisted them to avoid the crisis and better understand that others are available if that info was understood through education. (REG023)

As an assessor it is my experience that dementia frequently goes undiagnosed. It is obvious that an inpatient referred to me for assessment has some form of dementia but there is no formal diagnosis. Usually by the time the patient is referred to me, the dementia is advanced, the individual beyond community care and requiring long-term care placement. (SUR009)

As these quotations indicate, the comments provided by participants referred to inadequacy of current rural dementia care services, highlighting the challenges of providing timely and appropriate care for individuals with dementia and their caregivers in these rural settings.

## DISCUSSION

While the availability of different services varied, with some reported as widely available and others not available at all, the alignment of existing services with key aspects of primary health care was low across all dimensions. Although the average ratings of availability and primary health care orientation were low, the full range of response options were endorsed across the majority of individual items and scales.

### Availability

Across all categories, the basic health services were more often available in contrast to dementia-specific services. Health promotion programs for dementia were mostly unavailable. Primary health care providers and services such as nurse practitioners, dementia screening, and multidisciplinary team assessment, were unavailable in the communities served by approximately half of the participants. In terms of postdiagnostic support, dementia-specific services were mostly unavailable (counseling and registries for individuals with dementia and caregivers, caregiver support groups). Many basic home care services were available, but not transportation to health care and respite on weekends and nights, which may be especially useful for individuals with dementia and their caregivers. Dementia-specific long-term care services such as special care units in nursing homes were widely unavailable.

### Primary Health Care Orientation of Dementia-Related Services

Of the 30 items across the 6 scales, only 11 had mean scores over 3.0 (range 3.2 to 3.9 on a scale of 1 to 5) and could be considered somewhat positive. Three of 5 coordinated care items were rated above 3.0. This finding may reflect the role of Home Care Assessors in care coordination, which could lead to greater awareness and confidence in this aspect of care. All comprehensiveness of care items had means above 3.0 (3.2–3.9) reflecting some access to professionals who could provide diagnosis, management, and multidisciplinary care. Four of 5 items related to information and education were rated below 3.0 (awareness about what to do and where to go, information for caregivers, dementia-specific training for health care providers, and public education to decrease stigma). All accessibility items were rated low including transportation to services and equal access to services regardless of geographic location. Similarly, all population orientation items were rated low with respect to primary health care, home care, long-term care, telehealth services, and support for caregivers. For quality of care, 3 of 5 items had means less than 3.0 (timely diagnosis, use of standardized care pathways, adequate monitoring of patient safety).

Taken together, the study findings suggest significant gaps in the support for individuals with dementia and their informal caregivers in rural Saskatchewan. Compared with previous studies, which focused on perceptions of barriers and issues in rural dementia care service delivery, the current study provides a more fine-grained picture of which specific services are available and their appropriateness for dementia. The study findings are consistent with our consultations with decision makers regarding gaps in rural dementia care (Dal Bello-Haas, Cammer, Morgan, Stewart, & Kosteniuk, 2014; Morgan, Kosteniuk, et al., 2011), and with available research from elsewhere in Canada and globally. An international review of support for dementia in rural areas (Szymczynska et al., 2011) concluded that key issues that need to be overcome to address the needs of people with dementia and their caregivers are insufficient training for health professionals, limited availability and accessibility of postdiagnostic services, and lack of evidence on effective strategies for improving access to high quality diagnosis. In Canada, rural family caregivers reported fewer service options for community-based dementia services than those in urban settings (Forbes et al., 2008) . A study of service provision in rural England (McDonald & Heath, 2009) identified gaps including patchy service provision, absence of training opportunities, and difficulties recruiting staff.

Three studies have examined service provision for dementia in rural Scotland. The major gaps in service provision reported by individuals with dementia and their caregivers (Innes, Blackstock, Mason, & Smith, 2005) were transportation, respite, support for informal caregivers, home care, and day care services. Underutilization of existing services was attributed to services being inappropriate. Formal care providers identified distance and lack of transport, cost of services for the user, lack of choice in services, and a shortage of skilled staff as challenges in dementia service delivery (Innes, Cox, Smith, & Mason, 2006). In a third study, which explored rural dementia services from the perspective of rural service organizations (Mason, Blackstock, Cox, Innes, & Smith, 2005), 72% of respondents reported that dementia service coverage was inadequate.

The consistency in findings across these studies highlights the very real challenges of rural service provision, which are compounded when attempting to provide specialized services to a small isolated clientele. In the current study most basic health care services were available but services designed specifically for individuals with dementia and their informal caregivers were limited. The need for formal dementia care services will only increase as shortages of family caregivers and volunteers increase due to aging rural communities and continued out-migration of younger family members (Skinner et al., 2012). Although rural communities are viewed as having strong social networks, the sustainability of these networks to care for aging populations has been questioned (Skinner et al., 2012). Weirsma and Denton (2013) found that the social network available to individuals with dementia in rural northern communities could be fragile and could not replace formal support systems.

The Alzheimer Society of Canada’s Rising Tide Report (Dudgeon, 2010) noted that leading models and concepts in dementia care should inform Canadian policy, including integrated care models that improve care coordination, and chronic disease prevention and management models that promote team-based, evidence-driven, and integrated care. Additional recommendations include improving care at every stage on the continuum, providing caregiver support, emphasizing early intervention, and strengthening the dementia workforce (Dudgeon, 2010). The majority of people with dementia receive care within a primary care system, thus supporting the primary care system in providing dementia care is key to improving outcomes for rural patients and caregivers (Boustani, Schubert, & Sennour, 2007). There is a need for models of integrated primary health care that are effective and sustainable in rural settings. The models should include strategies for operationalizing best practices in primary health care for dementia—such as timely diagnosis, collaborative care planning, and on-going monitoring and management (Aminzadeh et al., 2012).

Aminzadeh et al. (2012) suggests that improving community-based primary health care for dementia will require that each community reviews its local resources, identifies missing links in the web of services, and develops clear shared care protocols and pathways that maximize service coordination and use of local resources. Aminzadeh et al. recommend development of innovative ways of using specialist resources in primary health care for dementia, which may be especially important in rural settings. While outreach programs (Morgan et al., 2009) and technology such as telehealth (e.g., Morgan, Crossley, et al., 2011; O’Connell et al., 2014) can help to reduce travel burden for episodic dementia care, the focus should be on providing appropriate support and services in the community across all stages of the care continuum. Building capacity for appropriate community-based care in rural health care providers and programs will be essential to achieving this goal, and will require innovative strategies for providing specialist-to-primary care provider training and consultation.

## LIMITATIONS

Without direct contact with Home Care Assessors we cannot be certain about how many received the survey. If some Assessors were missed, or if our count of eligible participants included individuals who were on leave, the response rate could be higher than reported. While Assessors were identified as most knowledgeable about services in their region, the findings represent the perspective of one group, and the Assessors may have ranked services within their scope of practice higher than other services. Finally, some of the scales developed to assess alignment with primary health attributes had low internal consistency reliability, suggesting that the scale items are not measuring just one concept and that more development work should be conducted to improve psychometric properties of the scale.

## CONCLUSION

This study identified key gaps in service availability and quality across the continuum for rural people with dementia and their caregivers. The defining characteristics of rural settings, including small geographically dispersed populations, create challenges in providing specialized services. Future research is needed that involves working with rural service providers, health care professionals, family caregivers and individuals with dementia to build on the strengths of rural systems, and to identify innovative strategies for improving dementia services in rural settings.

## FUNDING

Financial support for this research was provided by the Canadian Institutes of Health Research (#ACH 93185) and the Saskatchewan Health Research Foundation (#2104) through an Applied Chair in Health Services and Policy Research Award to the first author.
